# Effects of Oral Nutrition Supplementation with or Without Multi-Domain Intervention Program on Cognitive Function and Overall Health in Older Adults: A Randomized Controlled Trial

**DOI:** 10.3390/nu17111941

**Published:** 2025-06-05

**Authors:** Hae-Jin Kang, Eun-Hye Lee, Seong-Hye Choi, So-Young Moon, Jee-Hyang Jeong, Yoo-Kyoung Park

**Affiliations:** 1 Department of Medical Nutrition, AgeTech-Service Convergence Major, Graduate School of East-West Medical Science, Kyung Hee University, Yongin 17104, Republic of Korea; jjjjj@khu.ac.kr; 2 Department of Neurology, Ewha Womans University School of Medicine, Seoul 07985, Republic of Korea; oldarim@naver.com; 3 Department of Neurology, Inha University College of Medicine, Incheon 22212, Republic of Korea; seonghye@inha.ac.kr; 4 Department of Neurology, Ajou University School of Medicine, Suwon 16499, Republic of Korea; symoon.bv@gmail.com

**Keywords:** mild cognitive impairment, dementia, multi-domain intervention program, oral nutrition supplementation, MIND diet

## Abstract

**Objectives:** The global rise in dementia prevalence is escalating the socioeconomic burden, prompting efforts in prevention and treatment. This study aimed to evaluate the effects of an 8-week oral nutrition supplement (ONS) program with or without a multi-domain intervention program (MIP) in patients with mild cognitive impairment. **Methods**: Forty-nine patients with mild cognitive impairment were divided into three groups: (1) ONS (ONS), (2) ONS+MIP (ONS+MIP), and (3) control (CON). Korean-style dementia prevention MIP was used in the ONS+MIP group. Two packs of ONS per day were provided to the ONS group during the intervention period. Cognitive function, nutritional evaluation, body composition analysis, and physical performance were measured. The paired *t*-test and one-way analysis of variance were used for statistical analyses. **Results**: A final analysis was performed on the final 46 participants. After intervention, the cognitive function test (Repeatable Battery for the Assessment of Neuropsychological Status, RBANS) scores of the ONS+MIP group were significantly increased compared to the baseline scores. However, no significant changes were observed in the ONS and CON groups. Eating behavior and food quality also improved in the ONS+MIP group, with a significant difference among the three groups. There was no significant change in body composition in all groups; however, grip strength (left hand), muscular endurance, and the total SPPB score improved in the ONS+MIP group. The total SPPB score decreased in the CON group. **Conclusions**: Along with ONS intake, intensive education and continuous monitoring through multi-domain interventions are important to improve cognitive function. Trial registration: Clinical Research Information Service identifier: KCT0007253.

## 1. Introduction

Approximately >55 million people worldwide live with dementia, with approximately 10 million newly diagnosed annually [1]. In South Korea, the prevalence of dementia in the population aged ≥65 years was 10.4% in 2021, with approximately 890,000 people living with dementia, and owing to the rapid aging of the population, the incidence of dementia is expected to increase rapidly and exceed 1.42 million by 2030. A high rate of dementia has a high impact on patients and their families, and the social and economic influence on society at large is significant. Financially, the total cost of dementia management in Korea was KRW 18.7 trillion in 2021, and it is projected to double, reaching approximately KRW 36.9 trillion by 2030 [2]. Therefore, the early diagnosis and treatment of dementia is not only an issue at the personal level but also a national issue.

In response, the Korean government implemented a policy called the “National Responsibility System of Dementia” in 2017. It includes case management, enhancing medical support for patients with dementia, lowering medical and convalescent costs, extending long-term care services to patients with mild dementia, and fostering a dementia-friendly environment for the prevention of dementia. In particular, in the case of Alzheimer’s disease, which is the most common form of dementia, there is currently no medication available to eliminate the causative factor or dramatically slow disease progression [3]. The available medications can only be used to alleviate symptoms.

Several studies have investigated the prevention and treatment of dementia. There are a number of nutritional intervention studies for older individuals at risk of dementia, ranging from the supplementation of nutrients related to cognitive function (vitamin B complex, w3 fatty acids, antioxidants, phosphatidylserine) to the intake of oral nutrition supplements (ONSs) containing nutrients related to brain function to multi-domain interventions, including dietary educations [4,5,6,7,8].

Calapi et al. [9] evaluated cognitive function in healthy older participants after 12 weeks of administration of Conigrape^®^, a Vitis vinifera-based dietary supplement containing high amounts of polyphenol compounds. The overall Repeatable Battery for the Assessment of Neuropsychological Status (RBANS) score in the Conigrape^®^ administration group significantly increased by 6.07 points, whereas the overall RBANS score in the control group showed no significant difference.

Based on studies conducted in 2010 and 2012, significant enhancements in the memory domain of the cognitive function test were noted in the experimental group that received Souvenaid^®^ for 12 and 24 weeks. Souvenaid is a comprehensive nutritional drink containing crucial precursors and supportive nutrients, including uridine monophosphate, choline, omega-3 fatty acids (eicosapentaenoic acid [EPA] and docosahexaenoic acid [DHA]), phospholipids, and various vitamins such as B vitamins, vitamin C, and vitamin E, which collaboratively enhance membrane formation and function in individuals with Alzheimer’s disease [10]. In both studies, in addition to enhanced cognitive function, the EPA and DHA levels in red blood cells were improved.

To maximize the preventive effects of dementia, there is a growing emphasis on the importance of multi-domain interventions by not only correcting one form of lifestyle but also controlling various factors, such as vascular risk factors, including cognitive training, physical activity, nutrition education, and motivation reinforcement education [11].

In a previous study conducted in Finland, 1260 older patients participated in a multi-domain intervention study (Finnish Geriatric Intervention Study to Prevent Cognitive Impairment and Disability [FINGER]), and the group that underwent multi-domain intervention (nutrition, exercise, cognition training, and vascular risk monitoring) showed significant improvement in cognitive function scores after 2 years of intervention [12]. In the FINGER study, dietary coaching was performed through a combination of individual counseling (three sessions) and group meetings (six sessions) by a research nutritionist to achieve a healthy, balanced diet based on the Nordic Nutrition Recommendations [12].

The first Korean global network of multi-domain dementia prevention trials (World Wide FINGERs), called the South Korea study to prevent cognitive impairment and protect brain health through lifestyle intervention in at-risk older people (SUPERBRAIN) study, successfully showed that the multi-domain intervention trial was also effective in Koreans [13]. In the trial, the nutritional intervention included nutritional education about the Mediterranean-Dietary Approaches to Stop Hypertension Intervention for Neurodegenerative Delay (MIND) diet, which helps prevent dementia, adjusted to a Korean-style recipe. In this study, we aimed to directly intervene with nutrition through the provision of ONS with or without a multi-domain intervention program (MIP) in patients with Alzheimer’s disease and mild cognitive impairment (MCI).

## 2. Methods

### 2.1. Participants and Duration

This study was conducted from October 2020 to November 2021, and participants with MCI or early-stage Alzheimer’s disease aged between 65 and 85 years participated in this study.

Individuals with Korean-Mini-Mental State Examination, 2nd Edition scores of 17–27, with Clinical Dementia Rating (CDR) scale scores of 0.5–1, with CDR Sum of Boxes scores of 0.5–5, with the presence of a caregiver (guardian) who could attend the interview and program with participants, and agreement to participate in this study were eligible.

The exclusion criteria were as follows: degenerative and organic brain diseases that can be major causes of cognitive decline, such as brain tumor, stroke, normal pressure hydrocephalus, Parkinson’s disease, Lewy body dementia, vascular dementia, and autoimmune encephalitis; infectious or metabolic diseases that may lead to cognitive impairment, such as neurosyphilis, acquired immunodeficiency syndrome dementia, vitamin B12 deficiency, folate deficiency, and hypothyroidism; major psychiatric illness; epilepsy with refractory seizures; acute disease state; chronic liver disease or chronic kidney disease; chronic pulmonary or cardiovascular disease; any medical condition that would prevent cooperation with the intervention as determined by the study physician; severe visual or hearing impairment that may interfere with cooperation in this study; illiteracy; inability to safely participate in exercise programs; simultaneous participation in other studies; and no agreement to participate in this study.

**Figure 1:**
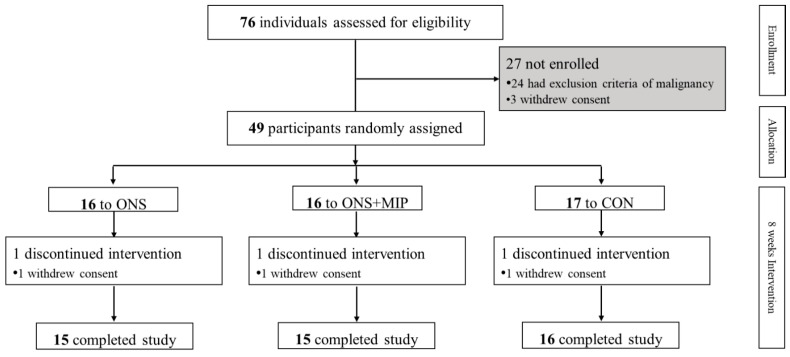
Diagram of study flow.

Of the 76 participants screened, 24 were excluded according to the exclusion criteria, and 3 withdrew their consent. Forty-nine participants were randomly assigned to three groups in a 1:1:1 ratio: ONS, only ONS group; ONS+MIP, ONS with MIP group; and CON, control group. Three participants, one from each group, dropped out, owing to the withdrawal of consent during the study, and data from 46 participants were analyzed (Figure 1).

### 2.2. Study Design

**Figure 2:**
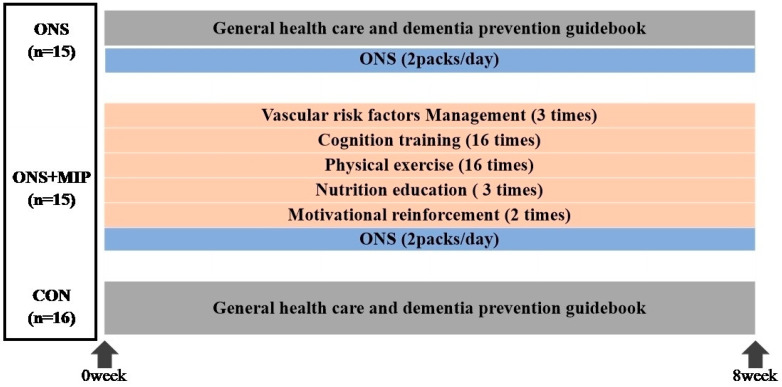
Study design.

This was an outcome assessor-blinded, single-center randomized controlled trial with a three-arm parallel design conducted over 8 weeks. The participants were randomized at baseline into two intervention and control groups by an independent statistician who used the permuted block randomization method with SAS macro programming (Version 9.4, SAS Institute, Cary, NC, USA). Only the specialist was aware of the full allocation sequence. The intervention group consisted of patients who received ONS alone and those who received ONS with the MIP (Figure 2). Participants in the control group were informed that they would be offered an MIP after the study. To ensure objectivity, outcome assessments and data analyses were independently conducted by evaluators who were blinded to group assignments and had no involvement in the intervention process. Assessments were carried out at baseline and after the 8-week intervention period. All participants were provided with information about the study by the researcher and gave written informed consent before participating in this study. Participants were informed that they could withdraw from the study at any time, and adverse events, including potential side effects, were continuously monitored throughout the intervention period. This study was approved by the Institutional Review Board of Ewha Womans University Seoul Hospital (SEUMC: 2020-08-008-010, approved: 26 January 2021).

#### 2.2.1. Intervention

ONS was formulated to include vitamins B6 and B12, folic acid, zinc, selenium, and nutrients associated with brain function, immunity, and antioxidant properties (Daesang Life Science, Seoul, Republic of Korea). Each pack provided 150 kcal/150 mL and a 5:2:3 ratio of carbohydrates, proteins, and fats. A summary of the nutrient composition is provided in Supplementary Table S1. Participants in the ONS and ONS+MIP groups consumed two packets per day.

The MIP, which is a modified version of SUPERBRAIN, was used in a previous study [13], and the participants received the intervention in individual sessions or in groups of two at the hospital. SUPERBRAIN is a Korean dementia prevention multi-domain intervention program consisting of five core components: nutrition counseling and education, vascular risk factors management, cognitive training, physical exercise, and motivational enhancement. Vascular risk factors were managed through consultations with physicians and nurses, who monitored key indicators such as blood pressure, waist circumference, smoking, and alcohol consumption. Pharmacological adjustments and lifestyle modifications were implemented when necessary to optimize vascular health. Cognitive training was conducted twice per week for 60 min, using tablet PCs and printed workbooks. The training addressed memory, executive function, visuospatial ability, and calculation skills through theme-based, engaging activities. Physical exercise was tailored to participants’ fitness levels based on baseline assessments and included both aerobic and resistance exercises. These sessions were led by certified exercise specialists and held twice per week, each lasting approximately 50 min. Nutrition education was provided by a clinical nutritionist across three sessions, focusing primarily on the Korean-adapted MIND diet and the general principles of healthy eating [14]. Motivational enhancement included group-based counseling sessions designed to foster emotional support and long-term behavioral change. Two structured group sessions were facilitated by professional counselors.

#### 2.2.2. Adherence

Adherence to the program was assessed using cumulative attendance rates for the 8-week intervention. Compliance with ONS was assessed by calculating the remaining packs after the end of study.

### 2.3. Outcome Measurements

#### 2.3.1. Repeatable Battery for the Assessment of Neuropsychological Status (RBANS) Total Scores

The primary outcome was the change in the total scale index score of the RBANS from baseline to the study end [15]. The RBANS is a simple tool administered by a nurse or occupational therapist at a medical institution, which takes approximately 30 min to complete and evaluates memory, visuospatial organization, language skills, attention, and memory recall. Higher scores indicate better cognitive function.

#### 2.3.2. Nutritional Assessments

Nutritional assessments were performed using the Nutrition Quotient for the Elderly (NQ-E) [16] and MIND diet checklists [14]. The NQ-E is a tool developed by the Korean Nutritional Society to determine the nutritional status of Korean seniors and consists of 19 questions about the frequency of daily nutrient intake, such as dairy products, eggs, beans, fish, vegetables, and fruits. Each question is divided into four detailed factors: “diversity”, “eating behavior”, “balance”, and “moderation”. Regarding the NQ-E results, the higher the score, the better the nutritional status. The MIND diet check sheet was created to check compliance with the MIND diet according to the composition and number of intakes of the MIND diet studied by Morris (2015) [14]. This checklist consists of 10 elements that must be consumed and five elements that must be avoided; the score is calculated as 1 point for each item, for a total of 15 points. The higher the score, the higher the dietary compliance.

#### 2.3.3. Body Composition and Physical Performance Assessments

For body composition and anthropometric measurements, height was measured using a stadiometer at the hospital. The body mass index (BMI) was calculated after measuring height and weight. Body fat (%), skeletal muscle mass (SMM), and visceral fat (VF) were measured using a body composition analyzer (InBody H20B, Biospace, Republic of Korea). Physical performance was evaluated using the Short Physical Performance Battery (SPPB), grip strength, and 30 s sit and standing test (endurance evaluation). The SPPB consists of three items: balance, walking speed, and standing up from a chair [17]. Balance was assessed by asking participants to maintain three standing positions (feet together, semi-tandem, and tandem) for up to 10 s each. Gait speed was measured as the time taken to walk a 4 m distance at a usual pace. The chair–stand test assessed lower body strength by recording the time required to complete five consecutive sit-to-stand repetitions from a chair without using the arms. Each SPPB item was scored from 0 (unable to perform) to 4 (best performance), with a total possible score of 12. Lower scores indicated poorer physical performance. Grip strength was measured using a digital hand dynamometer (CAMRY EH-101, Zhongshan Camry Electronic Co., Ltd., Zhongshan, China), and the average of two trials with the dominant hand was used. Additionally, the 30 s sit and standing test was administered to evaluate the muscular endurance of the lower limbs. Participants were instructed to rise to a full stand and return to a seated position as many times as possible within 30 s, and the total number of repetitions was recorded.

### 2.4. Statistical Analyses

The minimum sample size was calculated using G*Power (version 3.1.9.7, Düsseldorf, Germany), based on the expected effect size derived from a previous study [9], which reported a significant improvement in the RBANS total scale index scores (mean difference = 6.07, SD = 1.05 in the intervention group vs. mean difference = 0.48, SD = 5.30 in the control group). Based on the estimated effect size (f = 1.463), a significance level (α) of 0.01 (adjusted for multiple comparisons), and a statistical power (1 − β) of 0.80, a minimum of 13 participants per group was required. To account for an anticipated 15% dropout rate, 16 participants were enrolled in each group.

All statistical analyses were performed using IBM SPSS statistics version 28.0 (IBM Corporation, Armonk, NY, USA). The effectiveness evaluation targets were the per-protocol (PP) analysis results, which were adopted as the main analysis method [18]. PP analysis included only patients who strictly adhered to the protocol. However, since follow-up data were not available for participants who dropped out, an intention-to-treat (ITT) analysis could not be performed; if ITT had been applied, the overall effect size might have been reduced due to the inclusion of non-completers. Continuous variables were calculated using descriptive statistical analysis, and means and standard deviations were calculated. Categorical variables are expressed as n (%). Each variable was compared in terms of changes from weeks 0 to 8. Changes in the RBANS score, NQ-E, body composition, and physical fitness evaluation in each group were determined using the paired *t*-test. In some cases, tests were performed using the Wilcoxon signed-rank and Kruskal–Wallis tests. One-way analysis of variance (ANOVA) was performed to determine the differences among the three groups. All statistical analyses were verified for significance at a *p* < 0.05 level.

## 3. Results

### 3.1. Demographic Characteristics of the Participants

The demographic characteristics of the participants at baseline are shown in Table 1. Although the groups were randomly assigned, the proportion of women varied as follows: 33.3% in the ONS group, 60.0% in the ONS+MIP group, and 75.0% in the CON group. The ONS group included more men; however, the chi-squared test indicated that these differences were not statistically significant (*p* = 0.066).

### 3.2. Adherence Rates to the Multi-Domain Intervention Program and Oral Nutrition Supplement

The ONS adherence rates were 99.10% and 91.50% in the ONS+MIP and ONS groups, respectively. The adherence rate in the ONS+MIP group was higher than that in the ONS group, possibly due to the structured and diverse components of the intervention, which may have helped maintain engagement and promote consistent ONS intake.

The adherence rates for participants in the ONS+MIP group were 100% for vascular risk factor management, 96.05% for cognitive training, 94.00% for physical exercise, and 100% for nutrition education.

### 3.3. The RBANS Score

**Figure 3:**
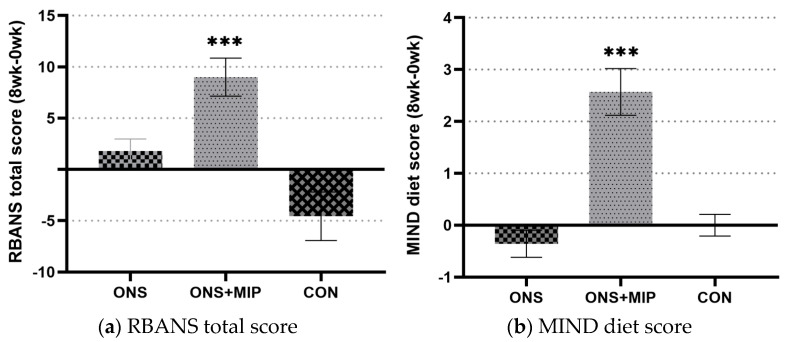
Changes and group differences in key outcomes after 8 weeks of intervention: (**a**) RBANS total score (cognitive function); (**b**) MIND diet score (dietary behavior). Values are expressed as mean change (8 wk−0 wk) ± SEM. Group sample sizes: ONS (*n* = 15), ONS+MIP (*n* = 15), and CON (*n* = 16). *** *p* < 0.001.

Cognitive function assessed using the RBANS was the primary outcome variable. Participants in the ONS+MIP group, who received ONS and MIP (SUPERBRAIN), showed significantly increased RBANS scores from 78.60 ± 16.89 to 87.60 ± 17.41 after 8 weeks (*p* < 0.001). Participants in the ONS group and the CON group showed some changes, which were not significant. As a result of comparing the changes in the RBANS total score among the groups, ONS+MIP had a significant difference compared with ONS and CON (*p* < 0.000) (Figure 3a).

### 3.4. The Mediterranean-Dietary Approaches to Stop Hypertension Diet Intervention for Neurodegenerative Delay (MIND) Diet Score

The MIND diet score is a measure of adherence to 10 recommended foods (foods to recommend for brain health) and five restricted foods (foods to avoid for brain health) [14]. Higher scores indicate a higher degree of practice. The changes in the MIND diet scores between the pre- and post-intervention periods are shown in Figure 3b. Participants in the ONS+MIP group had significantly increased MIND diet scores after 8 weeks (*p* < 0.000).

**Figure 4:**
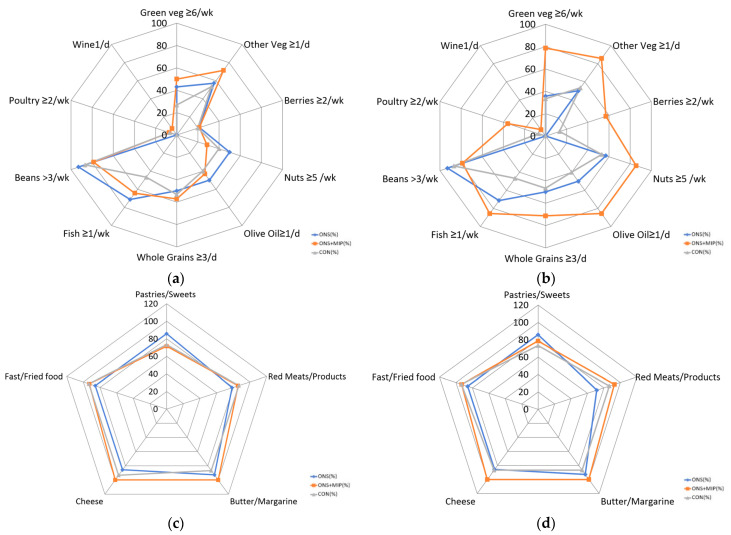
Changes in MIND diet components after 8 weeks of intervention: (**a**) foods to recommend for brain health (0 week); (**b**) foods to recommend for brain health (8 week); (**c**) foods to avoid for brain health (0 week); (**d**) foods to avoid for brain health (8 week). Group sample sizes: ONS (*n* = 15), ONS+MIP (*n* = 15), and CON (*n* = 16).

Figure 4 illustrates the changes in the MIND diet components before and after the 8-week intervention, with detailed results presented in Supplementary Table S2. Figure 4a,b show changes in the consumption of foods recommended for brain health, such as green leafy vegetables, nuts, whole grains, and fish. Meanwhile, Figure 4c,d depict changes in the intake of foods to avoid for brain health, including butter, cheese, processed meats, and fried foods. At baseline, no significant differences were observed among the three groups. However, following the intervention, participants in the ONS+MIP group demonstrated a significant increase in the consumption of brain-healthy foods and a reduction in the intake of foods detrimental to cognitive health. These findings suggest that integrating ONS with a multi-domain intervention (MIP) may have a synergistic effect in fostering positive dietary behavioral changes.

### 3.5. The Nutrition Quotient for the Elderly (NQ-E)

The NQ-E, which evaluates the nutritional status and quality of diet of seniors aged ≥65 years, was administered at baseline (week 0) and at the final week (week 8). Changes in the NQ-E total scores and sub-domain index (balance, diversity, moderation, dietary behavior) scores by group are shown in Table 2.

Participants in the ONS+MIP group showed a significant increase in the NQ-E scores after the intervention (*p* = 0.018), and this improvement was also significantly greater compared to the other groups (*p* = 0.002).

The sub-domain indices of the ONS+MIP group also increased, but only “moderation” significantly increased (*p* = 0.005). There were significant differences among the groups in the changes in the moderation (*p* = 0.001) and dietary behavior scores (*p* = 0.035).

### 3.6. Body Composition and Physical Performance

Changes in the body composition are shown in Table 3. Participants in the ONS+MIP and CON groups showed a slight increase in the BMI and SMM after 8 weeks, but there was no significant difference. No significant differences were observed among the groups when comparing the baseline and final body compositions.

Participants in the ONS+MIP group showed significantly improved grip strength (left hand) (*p* = 0.039), muscular endurance (*p* = 0.002), and SPPB total scores (*p* = 0.014). In the ONS group, other indicators increased, except for the SPPB score, but the differences were not significant. However, in the CON group, all items, except for left-hand grip strength, decreased; in particular, the SPPB total score significantly decreased after 8 weeks (*p* = 0.007). Post hoc tests among the three groups revealed significant differences between sitting and standing (*p* = 0.035) and total SPPB scores (*p* < 0.000).

### 3.7. Subgroup Analysis of RBANS, Short Physical Performance Battery, and NQ-E Change Scores According to Changes in the MIND Diet Score

Post hoc subgroup analysis was performed after classifying the participants according to their improvement in MIND diet scores as those who improved their MIND diet scores (Group A) and those who did not (Group B). There were 19 and 24 participants in Groups A and B, respectively. Participants in Group A showed improvements in their RBANS, SPPB, and NQ-E scores, whereas all participants in Group B showed a significant decrease in their scores (Table 4).

## 4. Discussion

In this study, we evaluated the effects of ONS with or without the MIP in older Korean adults with MCI or early-stage AD. Participants in the ONS+MIP group showed significant improvements in the RBANS total scores, which serve as a comprehensive measure of cognitive function; the NQ-E score, which reflects better nutritional status and diet quality; and the MIND diet score, which evaluates the level of nutrition education practice. In contrast, although the ONS and CON groups maintained or slightly improved in some measures, they did not exhibit significant changes across cognitive, physical, or behavioral outcomes. Body composition did not significantly change in any group, yet the ONS+MIP group demonstrated notable gains in physical performance measures, including grip strength and SPPB scores.

The ONS used in this study was formulated to provide essential nutrients critical for brain health, including vitamins B6 and B12, selenium, and zinc. These supplements offer a pragmatic approach to enhance nutrition for individuals experiencing cognitive impairment who may face challenges in meal preparation or consuming a balanced diet. The ONS aims to alleviate the meal preparation burden often faced by patients with dementia and their caregivers, thereby simplifying the process of achieving balanced nutrition [19]. Moreover, the ONS may indirectly support neuroprotection and cognitive function maintenance by elevating the blood levels of DHA and EPA, while reducing homocysteine levels [10]. However, as shown in this study, participants in the ONS group had a small but nonsignificant increase in the RBANS scores and no significant improvement in behavioral changes, such as the MIND diet scores. These findings suggest that while ONS provides essential nutrients, it alone may not be sufficient to induce cognitive or behavioral changes. Cognitive improvement typically requires active engagement through structured interventions involving cognitive stimulation, behavioral reinforcement, and motivational strategies. These results align with those of previous studies. For example, a study on folic acid supplementation in older adults with MCI showed significant biochemical improvements (e.g., serum folate and homocysteine) but limited cognitive benefits beyond specific domains [5]. Similarly, Scheltens et al. (2012) [10] found that Souvenaid improved memory-related test scores in patients with mild AD but did not lead to broader cognitive enhancement. Compared to these studies, our intervention period was shorter (8 weeks), and biomarkers were not measured, which may partly explain the limited effects of ONS alone. These findings collectively indicate that nutritional supplementation alone may have limited effects on cognitive outcomes unless combined with other intervention modalities.

In contrast, the ONS+MIP group in our study showed significant improvements in multiple domains—cognition, physical performance, and dietary behavior—highlighting the synergistic benefits of an integrated intervention approach. The multi-domain intervention, incorporating cognitive training, physical activity, nutrition education, and motivational enhancement, likely facilitated sustained behavioral changes and functional improvements. These findings were consistent with those of previous studies [12,13]. In a 2-year, multi-domain intervention (diet, exercise, cognitive training, vascular risk monitoring), randomized controlled trial [12] of 1260 older adults with cognitive decline in Finland, cognitive change, as measured by the Comprehensive NTB (Neuropsychological Testing Battery) Z-score, showed significant improvements in overall cognition, executive functioning, and processing speed, with significant improvements in the BMI, dietary habits, and physical activity. Moreover, the SUPERBRAIN [13] study in 136 South Korean older adults with at least one modifiable dementia risk factor resulted in significant improvements in the RBANS total score (*p* = 0.002) and NQ-E (*p* < 0.05). These results support the premise that multi-domain interventions addressing multiple risk factors are more effective in promoting cognitive and lifestyle improvements.

Participants in the ONS+MIP group also showed significant improvements in left grip strength, sit-and-stand, and SPPB total scores, which may be related to the lifestyle changes through multi-domain interventions, and improved muscle functions [20]. Patients with cognitive impairment and MCI may be chronically energy deficient because of reduced dietary intake [19]. The ONS provides these patients with convenient, high-concentration energy to help reduce fatigue during daily activities and replenish the energy required for physical activity [19]. The high adherence rate in the ONS+MIP group underscores the effectiveness and consistency of nutritional supplements, which positively affect physical functions, such as walking and balance, by reducing fatigue. Exercise intervention within the MIP seemingly contributed to improved muscle strength and increased activity levels through motivation and lifestyle adjustments, with ONS supplementation aiding in physical function improvements alongside the MIP. The SPPB is a comprehensive assessment of physical function that includes balance ability and walking speed in addition to muscle strength [17]; behavioral changes and improved muscle function may have played a role in the participants’ improved SPPB scores.

Despite these promising results, several limitations must be acknowledged. Although the sample size met the minimum requirement based on an a priori power analysis for the primary outcome (RBANS total scale index), the relatively small sample may have limited the ability to detect significant effects in secondary outcomes, such as physical performance and dietary behavior. Therefore, caution is warranted when interpreting these secondary results. Additionally, individual-level factors—such as educational attainment, baseline health status, and socioeconomic background—may have influenced the outcomes [21]. For instance, participants with higher education may have better understood and implemented intervention strategies. Subgroup analyses to control for these potential confounders were not performed. Moreover, although gender distribution was not statistically different among groups, the imbalance (e.g., 75% female in the CON group vs. 33% in the ONS group) may have affected the results, considering known sex-based differences in aging and response to interventions. Future studies should apply multivariate models to account for these covariates. Another limitation is the absence of long-term follow-up to assess whether the observed improvements are sustainable beyond the 8-week intervention period. Furthermore, the lack of biochemical marker assessments (e.g., serum folate and homocysteine) limits our understanding of the mechanistic pathways involved.

Nevertheless, a notable strength of this study was the high adherence rate to ONS. Both groups receiving ONS demonstrated adherence rates > 90%, with the ONS+MIP group achieving a 99.10% adherence rate and the ONS group achieving a 91.5% rate. Participants in the ONS+MIP group attended biweekly clinic visits for face-to-face interactions with the researchers, fostering program participation, ONS intake, and dietary habit monitoring. At each meeting, the researchers and therapists monitored program participation, ONS intake, and dietary habits and made recommendations. The favorable outcomes observed in the ONS+MIP group were likely due to the comprehensive and structured design of the intervention, which integrated multiple supportive components to promote participant engagement and adherence.

Second, this study demonstrated significant cognitive, behavioral, and physical improvements within a relatively short 8-week period, which contrasts with most previous trials requiring 12 to 24 weeks [9,10]. This suggests a potential synergistic effect of initiating ONS and the MIP simultaneously.

Lastly, this study underscores the potential of an integrated, lifestyle-based intervention combining nutrition, cognitive training, physical activity, and motivational support. Importantly, the exceptionally high adherence rate in the ONS+MIP group not only reflects strong participant engagement but also highlights the feasibility and acceptability of implementing such comprehensive interventions—including ONS—in real-world clinical and community settings for older adults with cognitive impairment.

## 5. Conclusions

This study demonstrates that integrating ONS with an MIP yields superior benefits compared to ONS alone in older Korean adults with MCI or early-stage AD. Participants in the ONS+MIP group exhibited consistent and clinically meaningful improvements across multi-domains, highlighting the synergistic effects of combining nutritional, cognitive, physical, and motivational strategies. The nutrient-dense and convenient formulation of the ONS used in this study is particularly advantageous for individuals experiencing difficulties in meal preparation or maintaining adequate dietary intake due to cognitive decline. While the ONS group demonstrated high compliance and modest improvements in nutritional status, it did not show significant changes in behavioral or functional outcomes, underscoring the limitations of standalone nutrition-focused interventions. These findings underscore the importance of a comprehensive, lifestyle-based approach to maximize intervention effectiveness.

For future research, expanding the sample size and including a broader range of participants would enhance the generalizability of findings. Additionally, incorporating objective biomarkers as outcome measures, conducting long-term follow-up or post-intervention assessments, and targeting nutritionally vulnerable populations—such as malnourished or socially isolated older adults—are warranted to further validate and extend the current results. Such approaches would offer deeper insights into the sustained effects and clinical applicability of multi-domain interventions in diverse real-world settings.

## Figures and Tables

**Table 1:** Demographic and clinical characteristics at baseline.

	ONS (*n* = 15)	ONS+MIP (*n* = 15)	CON (*n* = 16)	*p* Value ^(1)^
Female, *n* (%)	5 (33.3%)	9 (60.0%)	12 (75.0%)	0.066 ^(2)^
Age (year) *	76.8 ± 5.0	74.3 ± 5.5	76.0 ± 6.8	0.483
Education (year) *	11.4 ± 4.5	9.6 ± 3.6	8.5 ± 4.6	0.177
BMI (kg/m^2^) *	23.2 ± 2.2	23.0 ± 4.1	23.8 ± 1.9	0.729
RBANS *	74.7 ± 11.5	78.6 ± 16.9	79.5 ± 15.9	0.641
SPPB *	10.1 ± 1.3	9.3 ± 2.1	10.0 ± 1.9	0.468
NQ-E *	62.0 ± 9.1	56.5 ± 11.6	60.9 ± 9.9	0.312

BMI, body mass index; RBANS, Repeatable Battery for the Assessment of Neuropsychological Status; SPPB, Short Physical Performance Battery; NQ-E, Nutritional Quotient for the Elderly. * values are expressed as means ± standard deviations. ^(1)^ significantly different by one-way analysis of variance test with Scheffe’s post hoc test (*p* < 0.05). ^(2)^ significantly different by the chi-squared test with the Fisher–Freeman–Halton post hoc test (*p* < 0.05).

**Table 2:** Changes and group differences in the total scores of the NQ-E and sub-domain index scores after 8 weeks.

NQ-E		Group	Baseline	Week 8	*p*-Value ^(1)^	Δ8 w-0 w	*p*-Value ^(2)^
Total Scores		ONS (*n* = 15)	63.2 ± 8.06	62.8 ± 7.82	0.615	−0.5 ± 3.24	0.002 ** ^(4)^
		ONS+MIP (*n* = 15)	55.7 ± 11.50	62.0 ± 11.22	0.018 *	6.3 ± 8.71	
		CON (*n* = 16)	61.2 ± 10.13	61.1 ± 11.01	0.889 ^(3)^	−0.0 ± 2.93	
Sub-domain	Balance	ONS (*n* = 15)	56.0 ± 20.38	53.9 ± 20.95	0.498	−2.1 ± 11.40	0.527
		ONS+MIP (*n* = 15)	45.0 ± 22.74	52.6 ± 22.73	0.490	2.6 ± 13.84	
		CON (*n* = 16)	54.8 ± 13.17	54.5 ± 13.26	0.650	0.7 ± 7.32	
	Diversity	ONS (*n* = 15)	49.0 ± 16.88	48.0 ± 16.40	0.344 ^(3)^	−1.0 ± 3.36	0.053
		ONS+MIP (*n* = 15)	50.2 ± 12.90	52.4 ± 13.59	0.100	2.2 ± 4.61	
		CON (*n* = 16)	54.8 ± 13.17	54.5 ± 13.26	0.650	−0.3 ± 2.25	
	Moderation	ONS (*n* = 15)	77.9 ± 15.96	80.1 ± 12.40	0.264	2.2 ± 7.00	0.001 ** ^(5)^
		ONS+MIP (*n* = 15)	60.0 ± 13.05	75.8 ± 15.76	0.005 **	15.7 ± 17.33	
		CON (*n* = 16)	66.6 ± 21.68	67.3 ± 20.74	0.599	0.7 ± 4.84	
	Dietary behavior	ONS (*n* = 15)	62.8 ± 10.65	61.3 ± 11.40	0.165	−1.6 ± 3.99	0.035 *
		ONS+MIP (*n* = 15)	58.9 ± 14.30	60.8 ± 13.80	0.100	2.0 ± 4.15	
		CON (*n* = 16)	60.7 ± 14.28	59.60 ± 14.74	0.192	−1.1 ± 3.15	

NQ-E, Nutrition Quotient for the Elderly. ^(1)^ significantly different by paired *t*-test (*p* < 0.05), ^(2)^ significantly different by one-way analysis of variance (ANOVA) test with Scheffe’s post hoc test (*p* < 0.05), ^(3)^ significantly different by Wilcoxon-signed rank test (*p* < 0.05), ^(4)^ significantly different by Kruskal–Wallis test with Dunnett T3’s post hoc test (*p* < 0.05), and ^(5)^ significantly different by one-way ANOVA test with Dunnett T3’s post hoc test (*p* < 0.05). * *p* < 0.05 and ** *p* < 0.01.

**Table 3:** Changes and group differences in body composition and physical performance after 8 weeks.

Physical Status		Group	Baseline	Week 8	*p*-Value ^(1)^	Δ8 w–0 w	*p*-Value ^(2)^
Body composition	BMI (kg/m^2^)	ONS (*n* = 15)	23.0 ± 2.14	23.2 ± 2.10	0.424	0.2 ± 0.78	0.914
		ONS+MIP (*n* = 15)	23.0 ± 4.06	23.1 ± 4.17	0.454	0.2 ± 0.74	
		CON (*n* = 16)	23.8 ± 1.91	23.8 ± 1.69	0.773	0.1 ± 0.80	
	Body fat (%)	ONS (*n* = 15)	29.1 ± 7.19	29.7 ± 7.49	0.422	0.6 ± 2.55	0.620
		ONS+MIP (*n* = 15)	33.6 ± 9.66	33.4 ± 10.22	0.598	−0.3 ± 1.91	
		CON (*n* = 16)	34.2 ± 6.31	34.1 ± 6.18	0.924	−0.1 ± 2.56	
	SMM (kg)	ONS (*n* = 15)	22.2 ± 4.68	22.5 ± 4.80	0.355	0.2 ± 0.95	0.839
		ONS+MIP (*n* = 15)	20.2 ± 3.74	20.4 ± 3.58	0.397	0.1 ± 0.56	
		CON (*n* = 16)	20.1 ± 4.93	20.2 ± 4.72	0.641 ^(3)^	0.1 ± 0.81	
	VF (level)	ONS (*n* = 15)	7.4 ± 2.31	7.8 ± 2.67	0.139	0.4 ± 1.02	0.629
		ONS+MIP (*n* = 15)	9.5 ± 4.47	9.5 ± 4.47	1.000	0.0 ± 1.13	
		CON (*n* = 16)	9.3 ± 2.79	9.3 ± 2.59	1.000	0.0 ± 1.79	
Physical performance	Left grip (kg)	ONS (*n* = 15)	22.0 ± 7.42	23.1 ± 8.13	0.162	1.2 ± 2.89	0.396
		ONS+MIP (*n* = 15)	19.3 ± 7.17	20.7 ± 6.83	0.039 *	1.4 ± 2.22	
		CON (*n* = 16)	18.4 ± 8.24	18.6 ± 7.71	0.774	0.2 ± 2.45	
	Right grip (kg)	ONS (*n* = 15)	23.9 ± 7.28	24.2 ± 8.06	0.685	0.3 ± 2.39	0.313
		ONS+MIP (*n* = 15)	20.7 ± 7.10	21.9 ± 6.83	0.219	1.2 ± 3.44	
		CON (*n* = 16)	19.6 ± 7.66	19.3 ± 6.85	0.544	−0.4 ± 2.33	
	Sit and stand	ONS (*n* = 15)	12.5 ± 3.18	12.6 ± 5.20	0.888	0.1 ± 3.72	0.035 *
		ONS+MIP (*n* = 15)	11.9 ± 3.73	13.9 ± 3.53	0.002 **	1.9 ± 1.86	
		CON (*n* = 16)	13.1 ± 4.39	12.6 ± 4.27	0.185 ^(3)^	−0.4 ± 1.31	
	SPPB (total score)	ONS (*n* = 15)	10.1 ± 1.29	9.6 ± 1.34	0.131	−0.5 ± 1.16	<0.000 ***
		ONS+MIP (*n* = 15)	9.3 ± 2.16	10.9 ± 1.34	0.014 *	1.4 ± 1.78	
		CON (*n* = 16)	10.0 ± 1.90	9.1 ± 2.38	0.007 ** ^(3)^	−0.9 ± 1.00	

BMI, body mass index; SMM, skeletal muscle mass; VF, visceral fat; SPPB, Short Physical Performance Battery. ^(1)^ significantly different by paired *t*-test (*p* < 0.05), ^(2)^ significantly different by one-way analysis of variance (ANOVA) test with Scheffe’s post hoc test (*p* < 0.05), and ^(3)^ significantly different by Wilcoxon-signed rank test (*p* < 0.05). * *p* < 0.05, ** *p* < 0.01, and *** *p* < 0.001.

**Table 4:** Changes in the RBANS, SPPB, and NQ-E scores as the MIND diet score changes.

Δ8 w–0 w	Group A ^(1)^ (*n* = 19)	Group B ^(2)^ (*n* = 24)	*p* Value ^(3)^
RBANS change scores	6.0 ± 7.71	−0.7 ± 9.26	0.017 *
SPPB change scores	0.7 ± 2.02	−0.7 ± 0.95	0.008 **
NQ-E change scores	4.5 ± 7.98	−0.2 ± 3.41	0.026 *

RBANS, Repeatable Battery for the Assessment of Neuropsychological Status; SPPB, Short Physical Performance Battery; NQ-E, Nutrition Quotient for the Elderly. ^(1)^ MIND diet score change is Δ8 w–0 w ≥ 1. ^(2)^ MIND diet score change is Δ8 w–0 w < 1. ^(3)^ significantly different by *t*-test (*p* < 0.05). * *p* < 0.05 and ** *p* < 0.01.

## Data Availability

The dataset presented in this study is available on request from the corresponding author. The data contain sensitive personal information of elderly participants with cognitive impairment, and we did not obtain consent from participants for third-party data sharing.

## Supplementary Materials

The following supporting information can be downloaded at: https://www.mdpi.com/article/10.3390/nu17111941/s1, Table S1: ONS components; Table S2: Changes of MIND diet components and total scores after 8 weeks.

## Abbreviations

OMS	Oral Nutrition Supplement
MIP	Multi-Domain Intervention Program
RBANS	Repeatable Battery for the Assessment of Neuropsychological Status
MIND diet	Mediterranean-Dietary Approaches to Stop Hypertension Intervention for Neuro-Degenerative Delay
SUPERBRAIN study	the South Korea study to prevent cognitive impairment and protect BRAIN health through lifestyle intervention in at-risk older people study
MCI	Mild cognitive impairment
AD	Alzheimer’s disease

## References

[1] World Health Organization (2024). Fact Sheets-Dementia. https://www.who.int/news-room/fact-sheets/detail/dementia/.

[2] Ministry of Health and Welfare (2023). Korean Dementia Observatory 2022.

[3] Frisoni G.B., Molinuevo J.L., Altomare D., Carrera E., Barkhof F., Berkhof J., Delrieu J., Dubois B., Kivipelto M., Nordberg A. (2020). Precision prevention of Alzheimer’s and other dementias: Anticipating future needs in the control of risk factors and implementation of disease-modifying therapies. Alzheimer’s Dement..

[4] Bo Y., Zhang X., Wang Y., You J., Cui H., Zhu Y., Pang W., Liu W., Jiang Y., Lu Q. (2017). The n-3 polyunsaturated fatty acids supplementation improved the cognitive function in the Chinese elderly with mild cognitive impairment: A double-blind randomized controlled trial. Nutrients.

[5] Ma F., Wu T., Zhao J., Han F., Marseglia A., Liu H., Huang G. (2016). Effects of 6-month folic acid supplementation on cognitive function and blood biomarkers in mild cognitive impairment: A randomized controlled trial in China. J. Gerontol. Ser. A Biomed. Sci. Med. Sci..

[6] Cenacchi T., Bertoldin T., Farina C., Fiori M., Crepaldi G., Azzini C., Girardello R., Bagozzi B., Garuti R., Vivaldi P. (1993). Cognitive decline in the elderly: A double-blind, placebo-controlled multicenter study on efficacy of phosphatidylserine administration. Aging Clin. Exp. Res..

[7] Shah R.C., Kamphuis P.J., Leurgans S., Swinkels S.H., Sadowsky C.H., Bongers A., Rappaport S.A., Quinn J.F., Wieggers R.L., Scheltens P. (2013). The S-Connect study: Results from a randomized, controlled trial of Souvenaid in mild-to-moderate Alzheimer’s disease. Alzheimer’s Res. Ther..

[8] Salva A., Andrieu S., Fernandez E., Schiffrin E., Moulin J., Decarli B., Rojano-i-Luque X., Guigoz Y., Vellas B., Group T.N. (2011). Health and nutrition promotion program for patients with dementia (NutriAlz): Cluster randomized trial. J. Nutr. Health Aging.

[9] Calapai G., Bonina F., Bonina A., Rizza L., Mannucci C., Arcoraci V., Laganà G., Alibrandi A., Pollicino C., Inferrera S. (2017). A randomized, double-blinded, clinical trial on effects of a Vitis vinifera extract on cognitive function in healthy older adults. Front. Pharmacol..

[10] Scheltens P., Twisk J.W., Blesa R., Scarpini E., Von Arnim C.A., Bongers A., Harrison J., Swinkels S.H., Stam C.J., De Waal H. (2012). Efficacy of Souvenaid in mild Alzheimer’s disease: Results from a randomized, controlled trial. J. Alzheimer’s Dis..

[11] Kivipelto M., Mangialasche F., Ngandu T. (2018). Lifestyle interventions to prevent cognitive impairment, dementia and Alzheimer disease. Nat. Rev. Neurol..

[12] Ngandu T., Lehtisalo J., Solomon A., Levälahti E., Ahtiluoto S., Antikainen R., Bäckman L., Hänninen T., Jula A., Laatikainen T. (2015). A 2 year multidomain intervention of diet, exercise, cognitive training, and vascular risk monitoring versus control to prevent cognitive decline in at-risk elderly people (FINGER): A randomised controlled trial. Lancet.

[13] Moon S.Y., Hong C.H., Jeong J.H., Park Y.K., Na H.R., Song H.-S., Kim B.C., Park K.W., Park H.K., Choi M. (2021). Facility-based and home-based multidomain interventions including cognitive training, exercise, diet, vascular risk management, and motivation for older adults: A randomized controlled feasibility trial. Aging.

[14] Morris M.C., Tangney C.C., Wang Y., Sacks F.M., Bennett D.A., Aggarwal N.T. (2015). MIND diet associated with reduced incidence of Alzheimer’s disease. Alzheimer’s Dement..

[15] Randolph C., Tierney M.C., Mohr E., Chase T.N. (1998). The Repeatable Battery for the Assessment of Neuropsychological Status (RBANS): Preliminary clinical validity. J. Clin. Exp. Neuropsychol..

[16] Chung M.J., Kwak T.K., Kim H.Y., Kang M.H., Lee J.S., Chung H.R., Kwon S., Hwang J.Y., Choi Y.S. (2018). Development of NQ-E, Nutrition Quotient for Korean elderly: Item selection and validation of factor structure. J. Nutr. Health.

[17] Guralnik J.M., Ferrucci L., Simonsick E.M., Salive M.E., Wallace R.B. (1995). Lower-extremity function in persons over the age of 70 years as a predictor of subsequent disability. N. Engl. J. Med..

[18] Boutis K., Willan A. (2011). Intention-to-treat and per-protocol analysis. Cmaj.

[19] Lee J.-Y., Hyun Y.-S., Kim H.-S. (2019). Nutritional status of Korean elderly with dementia in a long-term care facility in Hongseong. Nutr. Res. Pract..

[20] Ikeuchi T., Kanda M., Kitamura H., Morikawa F., Toru S., Nishimura C., Kasuga K., Tokutake T., Takahashi T., Kuroha Y. (2022). Decreased circulating branched-chain amino acids are associated with development of Alzheimer’s disease in elderly individuals with mild cognitive impairment. Front. Nutr..

[21] Chen H., Dhana K., Huang Y., Huang L., Tao Y., Liu X., Van Lent D.M., Zheng Y., Ascherio A., Willett W. (2023). Association of the Mediterranean dietary approaches to stop hypertension intervention for neurodegenerative delay (MIND) diet with the risk of dementia. JAMA Psychiatry.

