# Supplementary material for: Effects of Oral Nutrition Supplementation with or Without Multi-Domain Intervention Program on Cognitive Function and Overall Health in Older Adults: A Randomized Controlled Trial
Source: Nutrients. 2025 Jun 5;17(11):1941. doi: 10.3390/nu17111941 (PMC12157949; doi:10.3390/nu17111941)
Supplement: Supplementary file 1 [file nutrients-17-01941-s001.zip › nutrients-3626978-supplementary.pdf]

# Supplementary tables

**Table S1. ONS components**

| Component (unit) | Amount (%)[1)] | Component (unit) | Amount (%)[1)] |
|---|---|---|---|
| Carbohydrates (g) | 20.5 (6%) | Vitamin B1 (mg) | 0.26 (22%) |
| Fat (g) | 6 (11%) | Vitamin B2 (mg) | 0.3 (21%) |
| Protein (g) | 5 (9%) | Vitamin B6 (mg) | 1.5 (100%) |
| Ca (mg) | 100 (14%) | Vitamin B12 (μg) | 3 (125%) |
| Phosphorus (mg) | 90 (13%) | Vitamin C (mg) | 40 (40%) |
| Potassium (mg) | 125 (4%) | Vitamin D (μg) | 1 (10%) |
| Magnesium (mg) | 25 (8%) | Vitamin E (mg $\alpha$-TE) | 20 (182%) |
| Sodium (mg) | 130 (7%) | Folate (μg) | 400 (40%) |
| Iron (mg) | 2.4 (20%) | Niacin (mgNE) | 15 (136%) |
| Zinc (mg) | 1.5 (18%) | Pantotenic acid (mg) | 1 (20%) |
| Iodine (μg) | 20 (13%) | Bioten (μg) | 6 (20%) |
| Vitamin A (μg RE) | 200 (29%) | Selenium (μg) | 60 (109%) |
| Other components[2)]: Eicosapentaenoic acid (EPA) and docosahexaenoic acid (DHA) containing fat, medium-chain triglycerides (MCT) oil, phosphatidyl serine, disodium 5'-uridylate | | | |

1) Ratio of content to daily nutritional standards (*based on* ENFORCEMENT RULE OF THE ACT ON LABELING AND ADVERTISING OF FOODS)
2) Information about the amount per serve is not available due to the policy of the Daesang Life Science Corporation that provided the products.
RE, retinol equivalent; NE, niacin equivalent; $\alpha$-TE, alpha tocopherol equivalent.

**Table S2. Changes of MIND diet components and total scores after 8 weeks**

| MIND diet | | ONS, N(%) | | ONS+MIP, N(%) | | CON, N(%) | |
|---|---|---|---|---|---|---|---|
| | | Baseline | Week8 | Baseline | Week8 | Baseline | Week8 |
| Total score | mean±SD | 8.6±1.55 | 8.3±1.77 | 8.8±1.67 | 11.4±1.91 | 8.1±1.98 | 8.1±1.83 |
| | p-value[1)] | 0.208 | | <0.000*** | | 1 | |
| components | Green Leafy ≥6/wk | 6(42.9) | 5(35.7) | 7(50.0) | 11(78.6) | 4(26.7) | 5(33.3) |
| | Other Vegetables ≥1/d | 8(57.1) | 7(50.0) | 10(71.4) | 12(85.7) | 8(53.3) | 8(53.3) |
| | Berries ≥2/wk | 3(21.4) | - | 3(21.4) | 8(57.1) | 3(20.0) | 2(13.3) |
| | Nuts ≥5 /wk | 7(50.0) | 8(57.1) | 4(28.6) | 12(85.7) | 6(40.0) | 8(53.3) |
| | Olive Oil≥1/d | 7(50.0) | 7(50.0) | 6(42.9) | 12(85.7) | 6(40.0) | 6(40.0) |
| | Whole Grains ≥3/d | 7(50.0) | 7(50.0) | 8(57.1) | 10(71.4) | 8(53.3) | 7(46.7) |
| | Fish ≥1/wk | 10(71.4) | 10(71.4) | 9(64.3) | 12(85.7) | 7(46.7) | 7(46.7) |
| | Beans >3/wk | 13(92.9) | 13(92.9) | 11(78.6) | 11(78.6) | 13(86.7) | 13(86.7) |
| | Poultry ≥2/wk | - | - | 1(7.1) | 5(35.7) | 1(6.7) | 1(6.7) |
| | Pastries, sweets <5/wk | 12(85.7) | 12(85.7) | 10(71.4) | 11(78.6) | 11(73.3) | 11(73.3) |
| | Red Meats and products<4/wk | 11(78.6) | 10(71.4) | 12(85.7) | 13(92.9) | 13(86.7) | 13(86.7) |
| | Butter, margarine <1T/d | 13(92.9) | 13(92.9) | 14(100.0) | 14(100.0) | 13(86.7) | 13(86.7) |
| | Cheese<1/wk | 12(85.7) | 12(85.7) | 14(100.0) | 14(100.0) | 14(93.3) | 13(86.7) |
| | Fast/fried food <1/wk | 12(85.7) | 12(85.7) | 13(92.9) | 13(92.9) | 14(93.3) | 14(93.3) |
| | Wine1/d | - | - | 1(7.1) | 1(7.1) | - | - |

MIND diet, the Mediterranean-Dietary Approaches to Stop Hypertension diet Intervention for Neuro-degenerative Delay diet

1) Significantly different by paired t-test (p<0.05)

***p<0.001
